# Comprehensive whole-genome resequencing unveils genetic diversity and selective signatures of the Xiangdong black goat

**DOI:** 10.3389/fgene.2024.1326828

**Published:** 2024-03-13

**Authors:** Ziao Liu, Haobang Li, Yang Luo, Jianbo Li, Ao Sun, Zulfiqar Ahmed, Baizhong Zhang, Chuzhao Lei, Kangle Yi

**Affiliations:** ^1^ Hunan Institute of Animal and Veterinary Science, Changsha, China; ^2^ Key Laboratory of Animal Genetics, Breeding and Reproduction of Shaanxi Province, College of Animal Science and Technology, Northwest A and F University, Xianyang, China; ^3^ Faculty of Veterinary and Animal Sciences, University of Poonch Rawalakot, Rawalakot, Pakistan

**Keywords:** population structure, selective signal, whole-genome resequencing, Xiangdong black goat, genetic diversity

## Abstract

Xiangdong black goats, indigenous to Hunan Province, China, exhibit remarkable adaptation to challenging environments and possess distinct black coat coloration alongside exceptional meat quality attributes. Despite their significance, comprehensive genomic investigations of this breed have been notably lacking. This study involved a comprehensive examination of population structure, genomic diversity, and regions of selection in Xiangdong black goats utilizing whole-genome sequencing data from 20 samples of this breed and 139 published samples from six other Chinese goat breeds. Our genomic analysis revealed a total of 19,133,125 biallelic single nucleotide polymorphisms (SNPs) within the Xiangdong black goat genome, primarily located in intergenic and intronic regions. Population structure analysis indicated that, compared with Jintang, Guizhou and Chengdu goats, Xiangdong black goats exhibit a reduced level of genetic differentiation but exhibit relatively greater divergence from Jining goats. An examination of genetic diversity within Xiangdong black goats revealed a moderate level of diversity, minimal inbreeding, and a substantial effective population size, which are more reflective of random mating patterns than other Chinese goat breeds. Additionally, we applied four distinct selective sweep methods, namely, the composite likelihood ratio (CLR), fixation index (*F*_ST_), *θ*_π_ ratio and cross-population extended haplotype homozygosity (XP-EHH), to identify genomic regions under positive selection and genes associated with fundamental biological processes. The most prominent candidate genes identified in this study are involved in crucial aspects of goat life, including reproduction (*CCSER1*, *PDGFRB*, *IFT88*, *LRP1B*, *STAG1*, and *SDCCAG8*), immunity (*DOCK8*, *IL1R1*, and *IL7*), lactation and milk production (*SPP1*, *TLL1*, and *ERBB4*), hair growth (*CHRM2*, *SDC1*, *ITCH*, and *FGF12*), and thermoregulation (*PDE10A*). In summary, our research contributes valuable insights into the genomic characteristics of the Xiangdong black goat, underscoring its importance and utility in future breeding programs and conservation initiatives within the field of animal breeding and genetics.

## 1 Introduction

Goats have a remarkable domestication history dating back approximately 10,000 years, making them one of the earliest domesticated animals (Monteiro et al., 2017). Goats are unique because of their excellent adaptability to a wide range of environments worldwide (Aziz, 2010). These animals play a crucial role in human society, providing not only meat and milk but also fiber, fertilizer, and even serve as a draft animal (Sinn and Rudenberg, 1992). The global goat diversity is astounding, with more than 557 different registered breeds and a population of 1.006 billion as of 2022 estimates (Kim et al., 2019; Wang et al., 2016). Goats inhabit every continent of the world except Antarctica and thrive in environments as diverse as tropical rainforests, hot deserts, and high-altitude regions (Hirst, 2019). Remarkably, goats not only survive but efficiently reproduce under extreme conditions, making them a top choice for farmers with limited resources.

In China, where the landscape varies from the northern pastoral region to the Qinghai–Tibet Plateau, mixed pastoral-agricultural regions, and northern and southern agricultural regions, there are approximately 60 native domestic goat breeds (Ministry of Agriculture and Rural Affairs of the People’s Republic of China, 2021). In recent years, these domestic breeds have garnered significant attention as crucial genetic resources. Consequently, the conservation of domestic animal diversity has become essential for meeting future needs. Recognizing their unique economic and ecological characteristics, China has initiated conservation programs. These initiatives include the establishment of conservation areas, conservation farms, and gene banks dedicated to preserving genetic resources of special breeds to safeguard indigenous animal populations for the benefit of future generations (Wei et al., 2014).

The Xiangdong black goats, native to Hunan Province in China, are a distinct indigenous breed known for their black hairy coat. These goats excel in adapting to challenging environments and can thrive on coarser feed. They are primarily raised for their meat, which is renowned for its low odor, high lean content, delicious flavor, and exceptional nutritional value (Mao-Liang et al., 2016; Chen Y M et al., 2022). The literature on complete genomic architecture, including population structure, genome diversity and evolutionary selective sweeps, in relation to this important genetic resource is scant. Earlier, studies relied on mapping of the mitochondrial genome (Mao-Liang et al., 2016) and elucidating single-gene associations with growth traits (Chen Y et al., 2022).

To address this substantial research gap, we conducted whole-genome resequencing on twenty Xiangdong goats. Furthermore, we included the whole-genome data of 139 goats from six different populations across China for subsequent analysis. This dataset, of 159 goat whole-genome sequences was used to investigate comparative phylogeny, genome diversity, and shared selection signals specific to the Xiangdong black goat. This pioneering study has the potential to significantly contribute to the utilization of germplasm resources of this breed in future breeding programs.

## 2 Materials and methods

### 2.1 Ethics statement

The study received approval from the Institutional Animal Care and Use Committee of Northwest A&F University (permit number: NWAFAC1019) in accordance with the regulations governing experimental animals in China. Specific consent procedures were not necessary, as we adhered to the National Standard for the Care and Use of Laboratory Animals to ensure the animals’ wellbeing and safety.

### 2.2 Animal sampling and genomic DNA extraction

**FIGURE 1:**
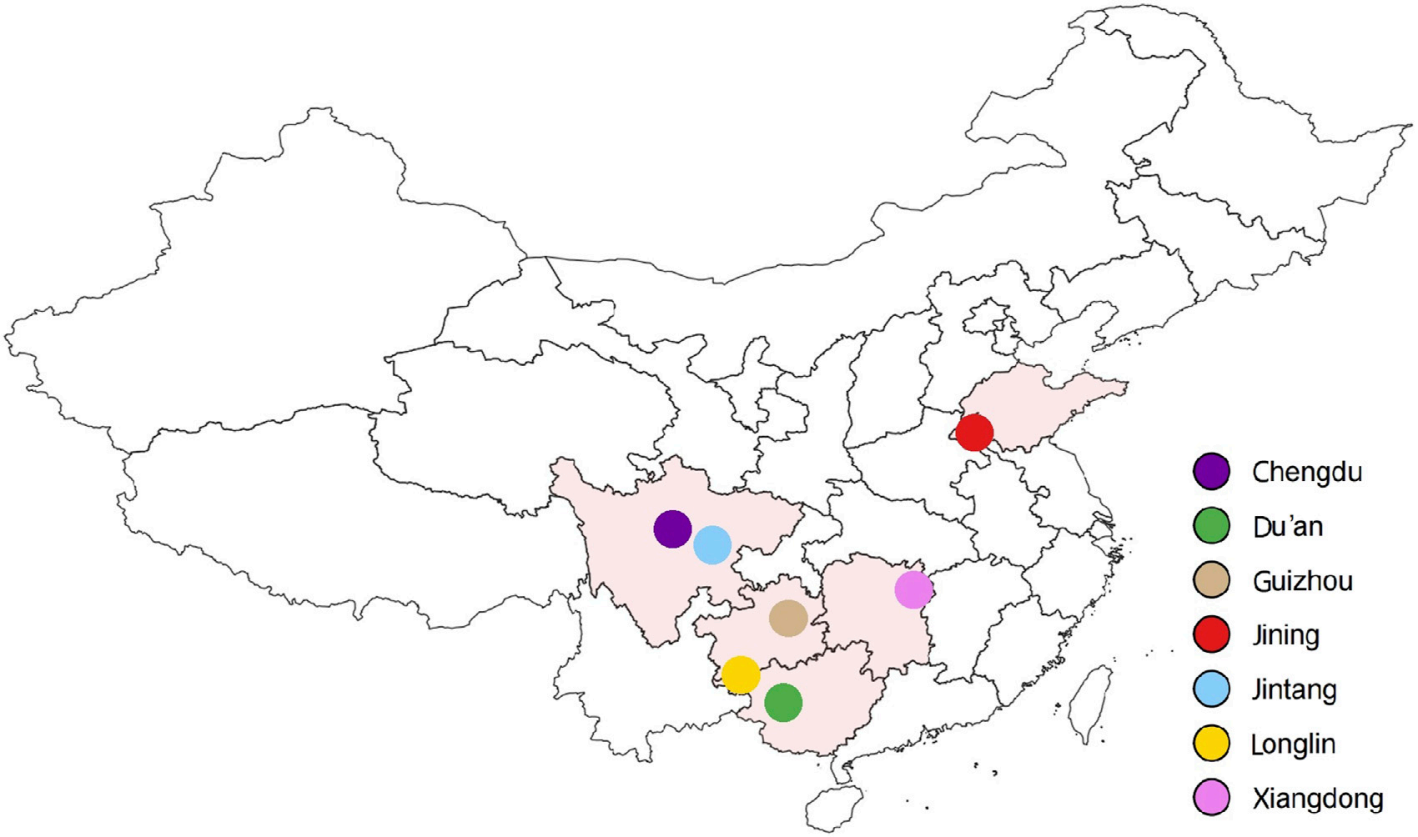
Sample geographic distribution of the 159 goats included in this study. [Xiangdong black goat (*n* = 20), Jintang goat (*n* = 45), Jining goat (*n* = 37), Chengdu goat (*n* = 15), Guizhou goat (*n* = 5), Longlin goat (*n* = 20), and Du’an goat (*n* = 17)].

The ear tissue of 20 pure unrelated adult Xiangdong black goats was collected from core breeding tracts in Hunan Province, China. These samples were screened based on the criteria of being between 2–8 years old, having a benign physical condition, with bright fur, normal body size, and uniform body shape. The tissue samples were preserved in 75% ethanol prior to being transferred to the laboratory. Genomic DNA was isolated from tissue samples by standard phenol–chloroform method as previously described (Sambrook et al., 1983). Additionally, publicly available data on 139 goat breeds from six distinct populations in China, namely, the Jintang (*n* = 45), Jining (*n* = 37), Guizhou (*n* = 5), Du’an (*n* = 17), Chengdu (*n* = 15) and Longlin (*n* = 20) breeds, were obtained from the Sequence Read Archive (SRA, https://www.ncbi.nlm.nih.gov/sra; Xiangdong goat: BioProject: PRJNA1028374, https://dataview.ncbi.nlm.nih.gov/object/PRJNA1028374?reviewer=ilnadkedc9m4jfuo6mfvkv5cuj; Guizhou goat: BioProject: PRJNA1027075, https://dataview.ncbi.nlm.nih.gov/object/PRJNA1027075?reviewer=mreaulgf2cms3a464mpob1u00e) database (Leinonen et al., 2010) (Figure 1). The details of each sample used in the dataset are presented in Supplementary Table S1.

### 2.3 Library construction and sequencing

The paired-end libraries were constructed using the TruSeq Library Construction Kit from Illumina, United States, in accordance with the manufacturer’s guidelines. Notably, each library was prepared with approximately 500 ng of genomic DNA, possessing an average fragment length of 70 kilobases. The average insert size for each library was 500 bp with average read length of 150 bp was assembled for each sample to be sequenced. The Illumina NovaSeq 2500 platform was used for sequencing, while the Novogene Bioinformatics Institute in Shenzhen, China, managed the entire operation.

### 2.4 Mapping and SNP calling

The raw reads were cleaned using Trimmometic v.0.39 software with default parameters, “LEADING:20 TRAILING:20 SLIDINGWINDOW:3:15 AVGQUAL:20 MINLEN:35 TOPHRED33” from sequencing artifacts and low-quality reads (Bolger et al., 2014). The clean reads were mapped to the goat reference assembly (GCA_001704415.2) using the default parameters of BWA-MEM (v.0.7.13-r1126) (Abuín et al., 2015). SAMtools was used to sort Bam files and “MarkDuplicates” from Picard v.2.20.2 for identification of potential PCR duplicates for the mapped reads, after which the reads were sorted through Picard software (McKenna et al., 2010). GVCF files were created using “HaplotypeCaller” in Genome Analysis Toolkit v3.8–1-0-gf15c1c3ef (GATK) with the “-ERC GVCF” option for SNP identification (McKenna et al., 2010). Then, “GenotypeGVCFs” and “SelectVariants” were used to call potential SNPs from the combined GVCF files. To ensure accuracy, GATK’s variant filtration was applied with settings such as “-cluster size 3′ and ”-cluster window 10′ to prevent false positives. The information of the sex chromosome clock is filtered out, retaining the genetic information in the autosomes. The specific criteria used for SNP filtering included a mean depth <1/3 and >3, quality by depth (QD) < 2, strand odds ratio (SOR) > 3, Fisher strand (FS) > 60, mapping quality (MQ) < 40, mapping quality rank sum test (MQRankSum) < −12.5, and read position rank sum test (ReadPosRankSum) < −8. Nonbiallelic SNPs and those with more than 10% missing genotype rates were removed. The remaining SNPs were annotated with a reference goat assembly based on their positions using ANNOVAR v2016- 02–01 (Danecek et al., 2011).

### 2.5 Analysis of genome diversity

To determine nucleotide diversity in each goat breed, we employed a sliding window method and VCFtools v4.1 software (Zhang et al., 2019). VCFtools (Danecek et al., 2011) was used to estimate the nucleotide diversity (*θ*_π_) of each breed, keeping a window size of 50 kb and a step size of 20 kb. Linkage disequilibrium (LD) was assessed using the *r* ^2^ statistic and PopLDdecay v3.40 software (Hudson et al., 1992), which considered the physical distance between pairwise SNPs with default settings. Runs of homozygosity (ROH) were evaluated by VCFtools utilizing parameters such as a homozygous density of 50, a homozygous window at 3, and a homozygous window missing 5. Additionally, we categorized ROH into four distinct ranges (0.5–1 Mb, 1–2 Mb, 2–4 Mb, and >4 Mb), and the results of LD and ROH analyses were visualized using R 3.6.1 software (Chan, 2018).

### 2.6 Population genetic analysis

A neighbor-joining (NJ) tree of all the samples was constructed using MEGA v7.0 software based on the genetic distance matrix provided by PLINK v1.9 (Vipan Kumar Sohpal et al., 2010; Purcell, S et al., 2007). The NJ tree was visualized using iTOL (Wang et al., 2010). Principal component analysis (PCA) was performed using smartPCA in EIGENSOFT v6.1 (Danecek et al., 2011). In addition, population structure was assessed using ADMIXTURE v1.3.0 with the default setting (Zhang et al., 2019). The number of assumed ancestry populations K ranged from 2 to 7.

### 2.7 Detection of selective sweeps

The cross-population selective sweeps in the Xiangdong black goat were determined by placing the Xiangdong black goat population as the target, and other goat populations in the dataset were placed in the reference group. Selective sweep analysis was performed through nucleotide diversity (*θ*_π_ ratio), the fixation index (*F*_ST_) and cross-population extended haplotype homozygosity (XP-EHH). The *F*_ST_ and *θ*_π_ ratios were analyzed through VCFtools (Szpiech and Hernandez, 2014) using a sliding window of 50 kb and 20 kb increments, and the chosen approach ensured a comprehensive and detailed examination of the data. The XP-EHH calculation was performed with selscan v1.1 software (Nielsen et al., 2005), and a 50 kb window was calculated for the population to determine haplotype-based selective sweeps.

To assess selection signals in Xiangdong black goats, the composite likelihood ratio (CLR) statistic was applied. The CLR was calculated using SWEEPFINDER2 software (Chen et al., 2021). For CLR, the top 5% of signaling regions were regarded as candidate selection signals (with cutoff values CLR >72.78). For the *θ*_π_ ratio, *F*_ST_ and XP-EHH, the top 5% regions with the most significant differences (with cutoff values of *F*_ST_ > 0.258, *θ*_π_ ratio >0.24 and XP-EHH >2.45) were selected as the significance thresholds, and the genes of interest were considered candidate genes.

To obtain additional functional information about the selected candidate genes, enrichment analysis of the genes in the gene sets was performed. Kyoto Encyclopedia of Genes and Genomes (KEGG) pathway analysis was performed on the candidate genes using KOBAS 3.0 and DAVID Bioinformatics (Xie et al., 2011; Huang, D. W et al., 2009).

## 3 Results

### 3.1 Sequencing read depths and landscape of SNPs

**FIGURE 2:**
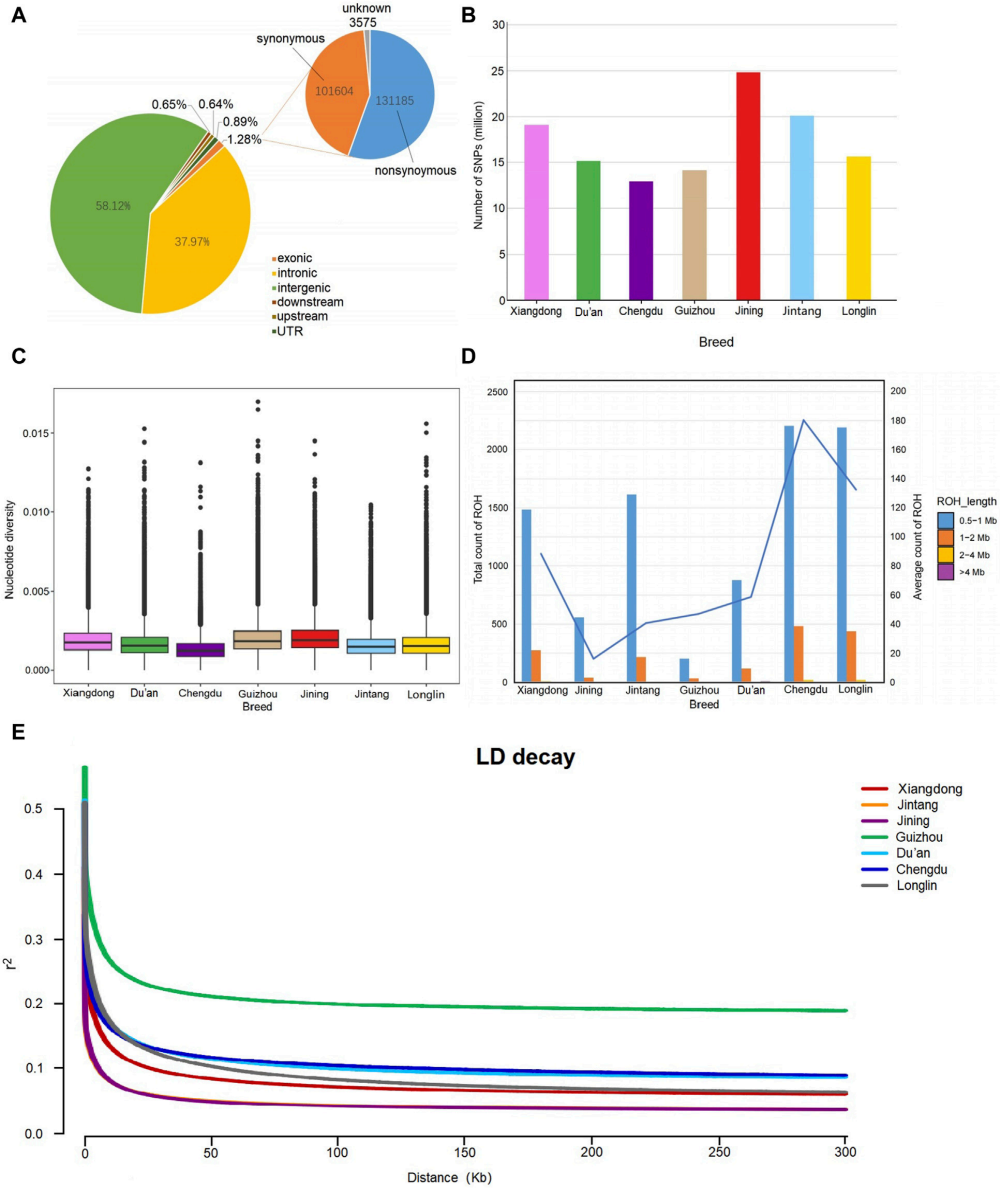
Genomic characteristics of the 7 goat breeds. **(A)** Functional annotation of the identified SNPs in Xiangdong black goats. **(B)** Number of identified SNPs in 7 goat breeds. **(C)** The box plot indicates the distribution of nucleotide diversity of each breed. **(D)** Counts of ROH individuals of the 7 breeds. The number represents the average number of individual ROHs for each breed. **(E)** Linkage disequilibrium (LD) decay curves of 7 goat breeds (tip: the LD decay curves of Jintang and Jining have a partial coincidence).

After aligning the clean reads with the *Capra Hircus* reference genome (GCA_001704415.2), we obtained 4.08 million reads with an average mapping rate of 98.37% and coverage depth of 12.39× (Supplementary Table S2). Together with those of the Xiangdong black goat, the genomic data of the other 6 goat populations of 19,133,125 biallelic SNPs were screened largely in intergenic and intron regions (58.12% and 37.97%, respectively). Only 1.28% of all the SNPs were found in exons, including 113,185 nonsynonymous and 101,604 synonymous SNPs. Additionally, 0.89% of the SNPs were screened in untranslated regions, 0.65% were found in downstream regions, and 0.64% were found in upstream regions (Figure 2A). The SNP numbers of the other six breeds are also listed in Figure 2B, and detailed information can be found in Supplementary Table S4.

### 3.2 Assessment of genetic diversity

The results of the nucleotide diversity analysis are presented in Figure 2C. The Jining goat exhibited the highest diversity (0.002070), followed by the Guizhou goat (0.002024), while the Chengdu goat presented the lowest (0.001353). The Xiangdong black goat has a mediocre value (0.001928) close to those of Guizhou (0.001687), Du’an (0.001704) and Longlin goats (0.001687). The most abundant observed ROH length was in the range of 0.5–1 Mb. The Chengdu and Longlin goats had a greater ROH length than did the Xingdong black goat, while the shortest ROH length was detected in the Jintang and Guizhou goats. The Jintang goat has a moderate length of ROH, similar to that of the Xiangdong goat breed (Figure 2D). The linkage disequilibrium (LD) decay curves of the 7 goat breeds are shown in Figure 2E. LD analysis revealed that the highest average LD (r2) was found in Guizhou goats, whereas the lowest LD was observed in Xiangdong black goats, followed by Jintang, Longlin, Du’an and Chengdu goats (Figure 2E).

### 3.3 Population structure and relationships

**FIGURE 3:**
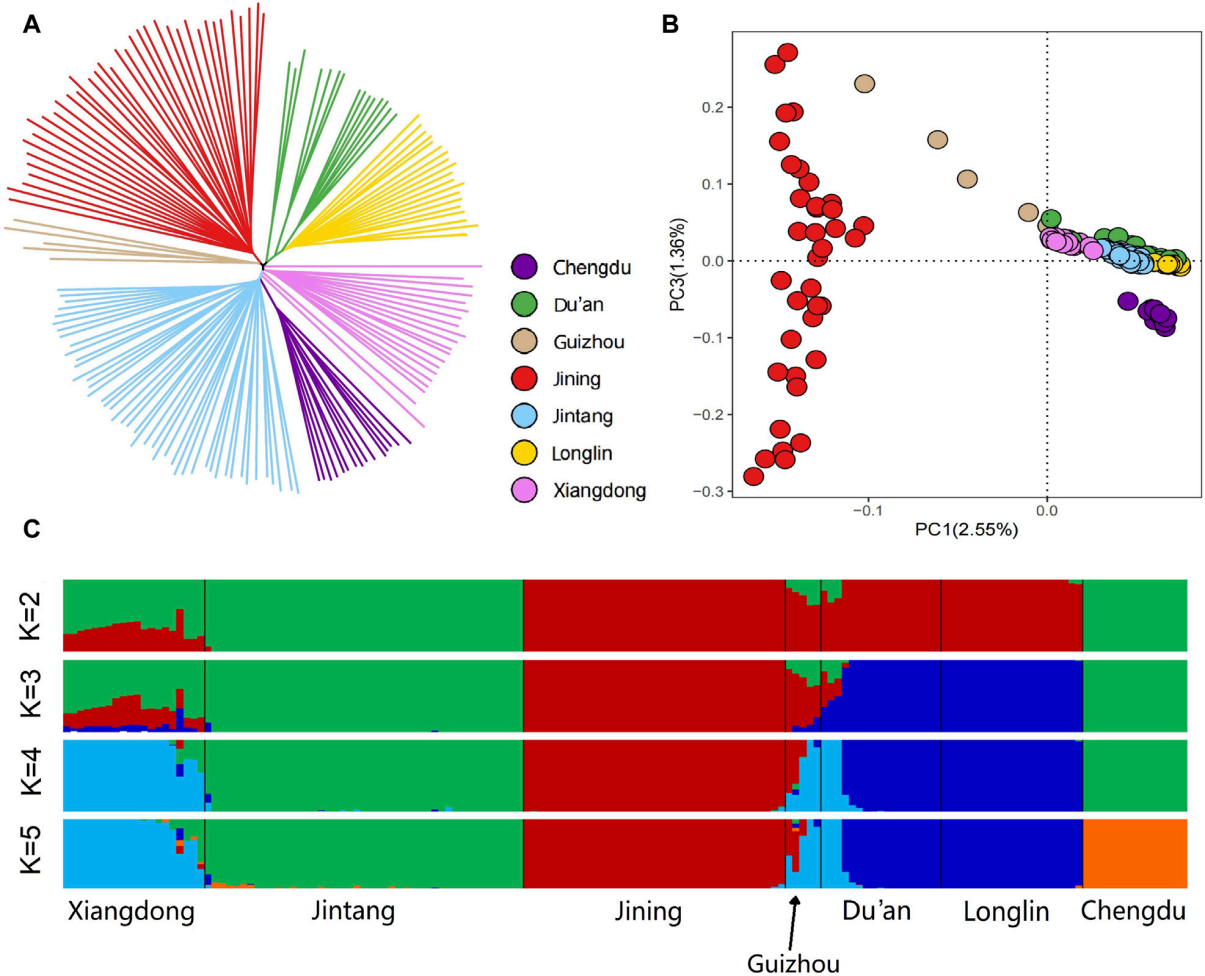
Genetic structure of the 7 goat breeds. **(A)** Neighbor-joining (NJ) tree of the 159 individuals based on the matrix of Hamming genetic distance. **(B)** Plants of the second principal component for the 159 individuals. **(C)** Ancestry proportions of each sample. Cross-validation error (CV) values were computed across groups, and the lowest CV value of 0.2349 was obtained at K = 3, which illustrates that K = 3 was the most likely number of genetically distinct groups within these 159 goat samples.

The NJ tree analysis revealed that each goat breed formed distinct clades. The individuals of each breed are distributed in clusters, and they are basically clustered on a branch without crossing each other. Notably, there was a significant difference between Xiangdong goats and Jining goats, which formed a relatively independent branch. In contrast, the Xiangdong goats were relatively close to the Jintang goats and Chengdu goats (Figure 3A). According to the autosomal-based PCA, PC1 accounted for 2.55% of the variation and effectively distinguished Jining goats from Xiangdong goats and the other breeds. The considerable distance between Xiangdong goats and Jining goats signifies a greater degree of differentiation between them. The distance between the other breeds and Jining goats was relatively great, and at the same time, the distance was closer to that of Xiangdong goats, indicating that the degree of differentiation between Xiangdong goats and the other five breeds of goats was less than that between Xiangdong goats and Jining goats (Figure 3B). Additionally, the ADMIXTURE analysis at K = 2 distinguished Jintang and Chengdu goats from Jining, Longlin, and Du’an goats, with Xiangdong and Guizhou exhibiting somewhat similar ancestral components. At K = 3, Xiangdong presented a similar admixture component to those of the Jintang, Guizhou, and Chengdu goat breeds. When K = 4, Xiangdong goats, Guizhou goats, and a portion of Du’an goats were distinguished from Jintang goats. Chengdu goat is distinguished from Jintang goat at K = 5. K = 3 yielded the lowest cross-validation error value and provided a meaningful representation of genetic differentiation (Figure 3C).

### 3.4 Selective signal analysis in the Xiangdong black goat

**FIGURE 4:**
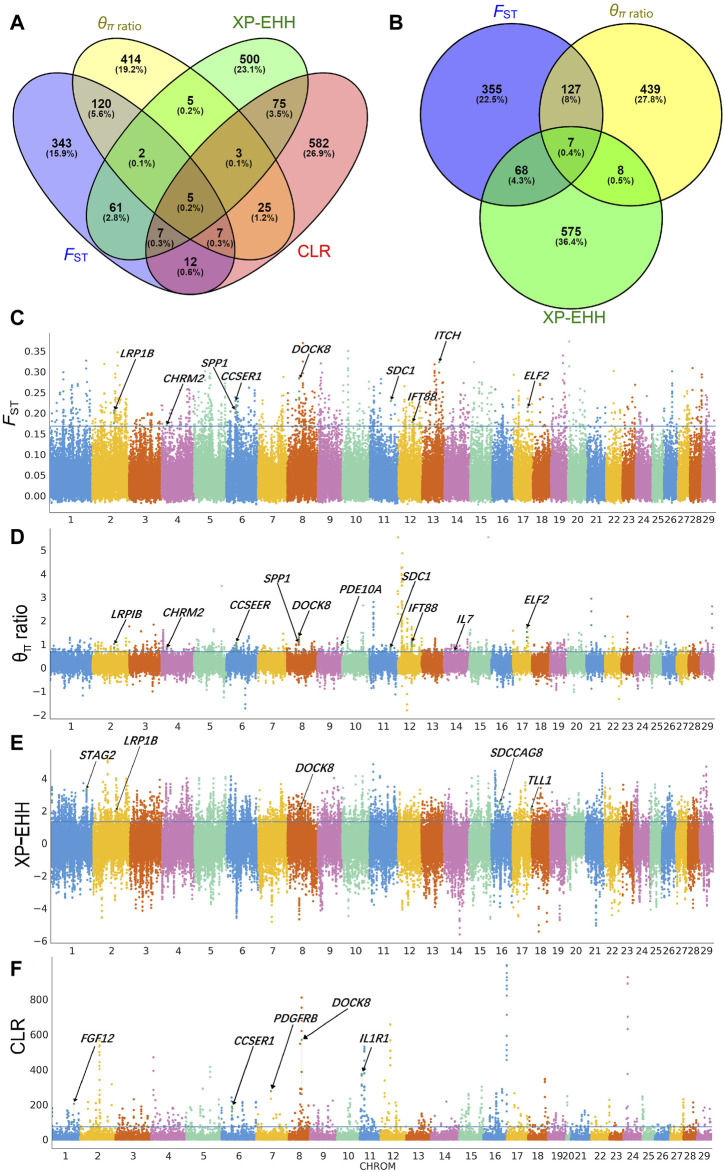
Identification of selective signals in Xiangdong goats. Overlapping genes according to four **(A)** or three **(B)** methods. Genome-wide distribution of *F*_ST_ **(C)**, the *θ*_π_ ratio **(D)**, XP-EHH **(E)**, and CLR **(F)**.

We used the CLR approach to investigate positive selective sweeps in the Xiangdong black goat. This led us to identify 716 genes as the top 5% of genes showing positive selection. When we examined sweeps across populations between the Xiangdong black goat and other goat breeds using the *F*_ST_, *θ*_π_ ratio, and XP-EHH methods, we identified 557, 581, and 658 genes, respectively, as the top signaling genes. Among these genes, 134 were found to overlap between the *F*_ST_ and *θ*_π_ ratio methods, 75 between the *F*_ST_ and XP-EHH methods, and 15 between the *θ*_π_ ratio and XP-EHH methods (Supplementary Table S3; Figures 4A, B). Furthermore, we discovered 138 genes associated with 12 significant gene ontology (GO) terms (Supplementary Table S3) (Table 1). The most significant GO term was related to the immune system, which responds to toxic substances. Other significant GO terms included five immune-related categories (positive regulation of T-helper 1 cell cytokine production, negative regulation of defense response to virus, immunological synapse formation, positive regulation of T-cell differentiation and positive regulation of B-cell differentiation), four growth- and development-related categories (GO terms related to growth factor activity, heart development, bone resorption and positive regulation of organ growth) and two mammogenesis-related categories (mammary gland epithelial cell differentiation and mammary gland alveolus development). The examination of overlapping genes identified through at least two selection methods revealed a set of genes relevant to important traits, such as reproduction (*CCSER1*, *PDGFRB*, *IFT88*, *LRP1B*, *STAG1*, and *SDCCAG8*), growth and development (*CCSER1, GALTNL6*), immunity (*DOCK8*, *IL1R1*, *IL7*), lactation and milk production (*SPP1*, *TLL1*, *ERBB4*), hair follicle/hair growth (*CHRM2*, *SDC1*, *ITCH*, and *FGF12*), and thermoregulation (*PDE10A*) (Figures 4C–F).

**TABLE 1:** Enriched GO terms among the putatively selected genes in the Xiangdong black goat.

Type	Name	Number of hits	Adjusted *p*-value
Cellular component	GO:0001771∼immunological synapse formation	12	0.005286
Cellular component	GO:0007507∼heart development	189	0.002701
Cellular component	GO:0060644∼mammary gland epithelial cell differentiation	13	0.005691
Cellular component	GO:0060749∼mammary gland alveolus development	16	0.006907
Biological process	GO:0009636∼response to toxic substance	82	0.000532
Biological process	GO:2000556∼positive regulation of T-helper 1 cell cytokine production	5	0.002443
Biological process	GO:0050687∼negative regulation of defense response to virus	11	0.00488
Biological process	GO:0045582∼positive regulation of T-cell differentiation	19	0.008121
Biological process	GO:0045579∼positive regulation of B-cell differentiation	1	0.005691
Biological process	GO:0046622∼positive regulation of organ growth	11	0.00488
Biological process	GO:0045453∼bone resorption	23	0.009738
Molecular function	GO:0008083∼growth factor activity	162	0.002003

## 4 Discussion

This study represents a comprehensive exploration of the genetic relationships, diversity, and positive selection signatures within Chinese goat populations. Whole-genome resequencing data encompassing a total of 159 goats were leveraged. Among these, 20 were newly sequenced Xiangdong goats, while the remaining 139 genomes were from previously published datasets. The primary objective of this investigation was to determine the genetic architecture underpinning the indigenous Chinese Xiangdong goat breed, marking its inaugural endeavor.

Studying genetic diversity plays an important role in the conservation and utilization of germplasm resources, the revelation of evolutionary history, and the investigation of phylogenetic relationships. The population genetic structure revealed that all the breeds in the dataset exhibited somewhat distinct clustering patterns. The results of the NJ-tree PCA and admixture analysis efficiently confirmed the population genomic characterization of Xiangdong and other contestant breeds. Due to the geographical location and different living environments, closed and natural mating conditions have existed for a long time, and as a result of the low intensity of artificial selection, these breeds are not very distinct from each other. However, breeds with closer geographic distributions shared common ancestral components and more genetic admixture. Similar observations were reported by (Xiong et al., 2023). Genetic diversity within livestock serves not only to delineate the genomic attributes of a given population but also as an indirect indicator of diverse evolutionary processes that have shaped it (Porto-Neto et al., 2013). The Xiangdong goat has a moderate level of genetic diversity, and the decreased LD decay and inbreeding levels revealed by ROH and effective population size make it a viable genetic resource. The monotonous environment and high degree of breeding may explain why the nucleotide diversity of the Xiangdong goat is lower than that of other Chinese goat breeds. This is attributed to the higher inbreeding coefficient and faster LD decay. Moreover, a greater level of nucleotide diversity and lower level of LD were found in the Jining goats, which was likely the result of gene flow between domestic and wild goats in Shandong, China. The highest nucleotide diversity may be related to population expansion or gene infiltration. In addition, a higher recombination rate is an indicator of a reliable state of genetic diversity (Hayes et al., 2003; Kijas et al., 2012). It is generally accepted that the greater the genetic diversity of a species is, the more adaptable its offspring will be (therefore, expansion is more likely to occur). Furthermore, low inbreeding indicates higher levels of heterozygosity, which are more linked to genetic variation and improved adaptability to harsh environmental situations (Makina et al., 2015a; Abondio et al., 2022). The average number of ROHs in Xiangdong goats is relatively high, which may provide a reference for the breeding and conservation of these goats since animals with high levels of ROH could be excluded or assigned lower priority for mating to minimize the loss of genetic diversity (Chen et al., 2020).

Positive selection represents a distinctive force that investigates alterations within the genome, offering insight into the evolutionary trajectory of a population. These alterations not only yield valuable insights into the specific genomic regions that undergo selection, whether through natural or artificial mechanisms but also provide a narrative of the underlying genetic processes that underpin the adaptation and advancement of a species (Hudson et al., 1992; Jeong and Di Rienzo, 2014; Werren et al., 2021). Within the realm of selection signal detection methods, CLR stands out for its ability to evaluate selection signals through comparisons between the likelihood of observing genetic data under a selection model and that under a neutral model (Chen et al., 2021). In contrast, XP-EHH employs a distinct approach, detecting selection signals through an examination of extended haplotype homozygosity differences between populations, thereby pinpointing regions characterized by unusually lengthy haplotypes in one population when contrasted with another. Moreover, the *F*_ST_ was harnessed to evaluate the reduced heterozygosity exhibited by the Xiangdong goat population in comparison to that of a reference population. This approach enabled the identification of regions characterized by distinct population attributes. Simultaneously, the *θ*_π_ ratio served to reveal the extent of divergence among different populations within the same species (Sabeti et al., 2002; Makina et al., 2015b).

Adapting to harsh environments relies on achieving the best possible growth and reproductive efficiency (Yang et al., 2019). In this study, we screened numerous genes related to reproduction and growth. Specifically, *PDGFRB* gene insertion, deletions and indels were associated with improved litter size in goats (E et al., 2019). Previously, the role of the *LRP1B* gene as a potential candidate gene for reproductive traits in Dazu black goats was revealed (Tao et al., 2021). Another gene, *SDCCAG8*, also serves as a potential regulator of reproductive phenomena, such as fecundity and litter size in goats (Seifi et al., 2021). In the case of body growth, *GALNTL6* was found to be among the top overlapping genes related to meat quality and carcass traits in chickens (Xu et al., 2023). Copy number variations in the *CCSER1* gene are linked to body growth traits, especially chest girth, in the Chinese goat population (Fu et al., 2020).

The natural habitat of the Xiangdong goat is the hilly terrenes of Hunan Province, China, where the environmental temperature may fluctuate throughout the year. Most often, cold conditions persist, and adapting to such inclement climatic conditions demands elevated hair growth and coat pigmentation. Many of the genes found through signal analysis, such as *CHRM2*, *SDC1*, *ITCH*, and *FGF12*, are related to hair follicle growth. Numerous genes were found to be the top signaling genes related to hair follicle and hair growth. Among these, the *IFT88* and *CHRM2* genes are regarded as candidate genes for mohair fineness in Tibetan cashmere goats (Bai et al., 2022), while the *SDC1* gene is associated with coarse and fine skin tissues of Liaoning cashmere goats (Nazari-Ghadikolaei et al., 2018). Similarly, the *ITCH* gene plays a potential role in regulating mohair and coat color traits in the Iranian Markhoz goat (Wang et al., 2021), whereas the *FGF12* gene is involved in the production of fleece in Inner Mongolia Cashmere goats (Chen Y et al., 2022).

In this study, we identified several important candidate genes related to immunity (*DOCK8*, *IL1R1*, and *IL7*). Among the immune response-related genes screened, *DOCK8* was investigated for its ability to confer immunity as a positively selected gene in Yunling goats (Kim et al., 2016). The *IL1R1* gene is regarded as a candidate gene for the induction of autoimmune responses in goats (Amiri Ghanatsaman et al., 2023); similarly, the *IL7* gene is the top signaling gene for the induction of immunological responses in the worldwide goat population (Crisà et al., 2016). Among milk production- and composition-related attributes, *SPP1* (Li et al., 2020), *TLL1* and *ERBB4*, which are related to colostrum and lactation regulation (Chen et al., 2020), respectively, have been studied previously. Additionally, genes involved in the response to environmental stressors and thermoregulation in the Du’an goat flock (Chen et al., 2020) were also found to be the most positively selected genes in this study.

## 5 Conclusion

In conclusion, for the first time, a comparative analysis of the Xiangdong black goat genome with those of other goat populations in China revealed genetic diversity, population genetic structure, and selection signals related to environmental adaptation-specific traits. Selective sweep analysis revealed several candidate genes associated with immunity, reproduction, growth and development, milk composition, hair and hair follicle growth, and thermoregulation. This study contributes significantly to animal breeding and genetics by enhancing the understanding of the genetic diversity and adaptation mechanisms of the Xiangdong goat breed to its native tracts.

## Data Availability

The datasets presented in this study can be found in online repositories. The names of the repository/repositories and accession number(s) can be found in the article/Supplementary Material.
